# Mesenchymal stromal cells in human immunodeficiency virus‐infected patients with discordant immune response: Early results of a phase I/II clinical trial

**DOI:** 10.1002/sctm.20-0213

**Published:** 2020-12-02

**Authors:** María Trujillo‐Rodríguez, Pompeyo Viciana, Inmaculada Rivas‐Jeremías, Ana I. Álvarez‐Ríos, Antonio Ruiz‐García, Olga Espinosa‐Ibáñez, Salvador Arias‐Santiago, Juliana Martínez‐Atienza, Rosario Mata, Olga Fernández‐López, Ezequiel Ruiz‐Mateos, Alicia Gutiérrez‐Valencia, Luis F. López‐Cortés

**Affiliations:** ^1^ Unidad Clínica Enfermedades Infecciosas, Microbiología y Medicina Preventiva, Hospital Universitario Virgen del Rocío/Instituto Biomedicina de Sevilla/CSIC/Universidad de Sevilla Avd. Manuel Siurto s/n SEVILLA España Spain; ^2^ Departamento de Bioquímica Clínica Hospital Universitario Virgen del Rocío/Consejo Superior de Investigaciones Científicas (CSIC)/Servicio Andaluz de Salud (SAS)/Universidad de Sevilla Seville Spain; ^3^ Unidad de Producción Celular e Ingeniería Tisular Complejo Hospitalario Universitario de Granada Granada Spain; ^4^ Red Andaluza en Diseño y Traslación de Terapias Avanzadas Fundación Pública Andaluza Progreso y Salud Seville Spain

**Keywords:** clinical trial, HIV infection, immunological nonresponders, mesenchymal stromal cells

## Abstract

Between 15% and 30% of HIV‐infected subjects fail to increase their CD4^+^ T‐cell counts despite continuous viral suppression (immunological nonresponders [INRs]). These subjects have a higher morbidity and mortality rate, but there are no effective treatments to reverse this situation so far. This study used data from an interrupted phase I/II clinical trial to evaluate safety and immune recovery after INRs were given four infusions, at baseline and at weeks 4, 8, and 20, with human allogeneic mesenchymal stromal cells from adipose tissue (Ad‐MSCs). Based on the study design, the first 5 out of 15 INRs recruited received unblinded Ad‐MSC infusions. They had a median CD4^+^ nadir count of 16/μL (range, 2‐180) and CD4^+^ count of 253 cells per microliter (171‐412) at baseline after 109 (54‐237) months on antiretroviral treatment and 69 (52‐91) months of continuous undetectable plasma HIV‐RNA. After a year of follow‐up, an independent committee recommended the suspension of the study because no increase of CD4^+^ T‐cell counts or CD4^+^/CD8^+^ ratios was observed. There were also no significant changes in the phenotype of different immunological lymphocyte subsets, percentages of natural killer cells, regulatory T cells, and dendritic cells, the inflammatory parameters analyzed, and cellular associated HIV‐DNA in peripheral blood mononuclear cells. Furthermore, three subjects suffered venous thrombosis events directly related to the Ad‐MSC infusions in the arms where the infusions were performed. Although the current study is based on a small sample of participants, the findings suggest that allogeneic Ad‐MSC infusions are not effective to improve immune recovery in INR patients or to reduce immune activation or inflammation. ClinicalTrials.gov identifier: NCT0229004. EudraCT number: 2014‐000307‐26.

## Graphical abstract

Between 15%‐30% of HIV‐infected subjects, do not achieve a significant immune recovery despite continuous viral suppression (immunological non‐responders). These subjects have an aberrant state of immune activation and inflammation. The treatment with adipose tissue allogeneic adult mesenchymal stromal cells does not neither improve immune recovery, nor has it succeeded in reducing the state of immune activation and inflammation.

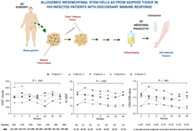

**Significance statement**

HIV infection is characterized by a progressive CD4^+^ T‐cell depletion and an immune overactivation and inflammation state. Antiretroviral therapy suppresses viral replication and reduces this aberrant state, leading to an immune recovery in a high proportion of subjects. However, between 15% and 30% of patients fail to achieve sufficient immune reconstitution, triggering a rise in the rate of morbidity associated with both AIDS and non‐AIDS events. These patients are called immune nonresponders (INRs), and no current therapies are available to treat them. Mesenchymal stromal cells have been shown to have immunomodulatory properties because they interact and modulate the function of multiple immune cells. For that reason, this clinical trial was planned to evaluate the efficacy and safety of infusions of human allogeneic mesenchymal stromal cells from adipose tissue (Ad‐MSCs) in INRs. However, results suggest that Ad‐MSC infusions are not effective to improve immune recovery in INRs or to reduce immune overactivation or inflammation state.

## Lessons learned

- Adipose tissue allogeneic adult mesenchymal stromal cells infusions are not effective to improve immune recovery or to reduce immune overactivation or inflammation state in immunologically nonresponding HIV‐infected patients.
- Donor‐associated factors and manufacturing procedure could affect efficacy and safety.

## Significance statement

HIV infection is characterized by a progressive CD4^+^ T‐cell depletion and an immune overactivation and inflammation state. Antiretroviral therapy suppresses viral replication and reduces this aberrant state, leading to an immune recovery in a high proportion of subjects. However, between 15% and 30% of patients fail to achieve sufficient immune reconstitution, triggering a rise in the rate of morbidity associated with both AIDS and non‐AIDS events. These patients are called immune nonresponders (INRs), and no current therapies are available to treat them. Mesenchymal stromal cells have been shown to have immunomodulatory properties because they interact and modulate the function of multiple immune cells. For that reason, this clinical trial was planned to evaluate the efficacy and safety of infusions of human allogeneic mesenchymal stromal cells from adipose tissue (Ad‐MSCs) in INRs. However, results suggest that Ad‐MSC infusions are not effective to improve immune recovery in INRs or to reduce immune overactivation or inflammation state.

## 1 INTRODUCTION

Chronic immune activation and inflammation is considered today as the main driving force of CD4^+^ T‐cell depletion and the functional impairment of the immune system caused by HIV infection.^1^ Antiretroviral therapy (ART) achieves the control of viremia in most subjects and reduces both cellular and soluble activation markers, leading to immune recovery in a high proportion of subjects.^2^ However, between 15% and 30% of subjects exhibit a poor CD4^+^ T‐cell recovery despite successful viral suppression; these subjects are known as immunological nonresponders (INRs).^3^ Although the definition of immunological nonresponse lacks consensus, it has always been based in the increase of CD4^+^ T‐cell counts above different thresholds in a given time period.^4^ These subjects show severe homeostatic alterations in CD4^+^ T cells, with a disturbed maturational profile, a reduced thymic function, and increased levels of activation and apoptosis, among other characteristics.^5^ From a clinical point of view, INRs show higher rates of morbidity and mortality associated with AIDS and non‐AIDS events such as cardiovascular events, neurocognitive impairment, non‐AIDS malignancies, end‐stage liver and renal diseases, bone disorders, and frailty than those with a good immune response.^6^, ^7^, ^8^ Moreover, when these subjects grow old, this situation will be aggravated by age‐associated immunosenescence.^9^ In these subjects, many strategies have been evaluated, such as ART intensification, immunomodulators, immunosuppressive agents, and probiotics, though with disappointing results; thus, no current effective therapies are available.^10^

On the other hand, several studies, both in vitro and in vivo, have shown that mesenchymal stromal cells (MSCs) can modulate the function of T helper cells and B lymphocytes, natural killer (NK) cells, and dendritic cells, whereas stimulating regulatory T (T_reg_) cells results in a change from a proinflammatory state to an anti‐inflammatory state.^11^, ^12^, ^13^ These properties have been demonstrated in multiple animal models of disease and have been used successfully in humans with graft vs host disease and several autoimmune and nonimmune diseases.^14^, ^15^ Up to now, only one study has been carried out with MSCs from cord blood in INRs, which resulted in a significant increase in circulating CD4^+^ T lymphocytes and a decrease of the activation of T lymphocytes and soluble inflammation mediator levels without significant adverse effects or loss of viremia control,^16^ but this study has not been replicated. Thus, our aim was to evaluate, for the first time, whether MSCs coming from a more accessible source, such as adipose tissue, are safe and effective in improving the immune recovery in INRs.

## 2 MATERIALS AND METHODS

### 2.1 Study design

This was originally planned as a phase I/II, randomized, placebo‐controlled, clinical trial designed to evaluate the safety and efficacy of adipose tissue allogeneic adult MCSs (Ad‐MSCs) in INRs. The design was carried out jointly with the Andalusian Network for the Design and Translation of Advanced Therapies (http://terapiasavanzadas.junta-andalucia.es), which also acted as sponsor.

Because of security concerns, in the first phase, five eligible INRs received unblinded Ad‐MSCs with a safety minimum period of 15 days between patients. Once all five subjects had completed the four Ad‐MSC infusions, an independent data‐monitoring committee (IDMC) performed a preliminary analysis of safety and efficacy data. In the second phase, (permanently suspended), 10 additional subjects would have been randomized to receive Ad‐MSC infusions or placebo.

### 2.2 Study approval

The clinical trial was approved by the National Health Authority and the Ethics Committee for Clinical Research of the participating site. All of the subjects provided informed consent. It was registered at the European Medicine Agency with EudraCT number 2014‐000307‐26 and Clinical trials.gov number NCT0229004, and it was conducted according to the principles of Good Clinical Practice (GCP) at Virgen del Rocío University Hospital in Seville (Spain).

### 2.3 Adipose tissue allogeneic adult mesenchymal stromal cells

Ad‐MSCs were provided by the Cell Production and Tissue Engineering Unit of Virgen de las Nieves University Hospital (Granada). Ad‐MSCs were obtained from subcutaneous adipose tissue samples from four donors obtained by surgical exeresis in aseptic conditions fulfilling the provisions of the Spanish Royal Decree 1301/2006.

The donors were younger than 60 years and negative for hepatitis B virus, hepatitis C virus, and HIV infection and *Treponema pallidum*. Only one donor was diabetic and a smoker (Table S1). Once the adipose tissue was obtained, a mechanical disintegration of the tissue was performed, followed by an enzymatic digestion with collagenase type A. The cell fraction was separated by centrifugation and seeded in plates, and after two culture‐expansion passages Ad‐MSCs were isolated. The formulation of medium for Ad‐MSC expansion was as follows: Dulbecco's modified Eagle's medium with 10% of fetal bovine serum, 2% of l‐alanine and l‐glutamine, 0.1 mg/mL of gentamicin, and 100 UI/mL of penicillin. After the expansion Ad‐MSCs were frozen and put into quarantine until quality controls were performed (Table S2). When a patient was included in the clinical trial, Ad‐MSCs were thawed and expanded for 1 week, and new quality controls were performed before delivery (Table S3). The finished product was a cell suspension containing allogeneic expanded Ad‐MSCs at a concentration of 2 000 000 cells per milliliter in Ringer's lactate solution containing 1% human albumin. The volume was adjusted according to the patient's weight and packaged in sterile bags preserved at 2°C to 8°C until their intravenous infusion at a dose of 1 × 10^6^ Ad‐MSCs per kilogram of body weight (Figures S1 and S2).

### 2.4 Cell infusion procedure

**FIGURE 1:**
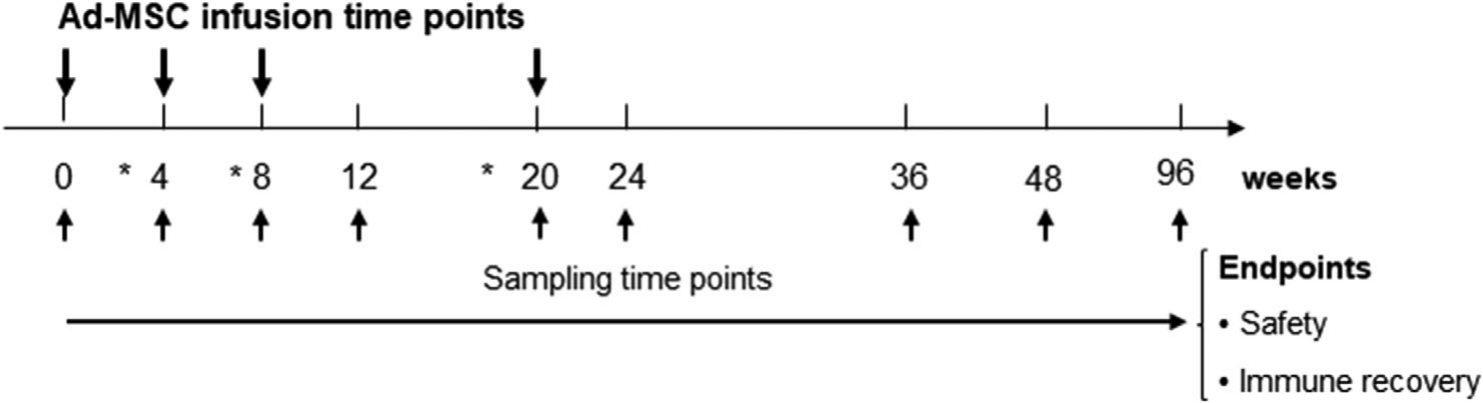
Scheme of visits, infusions of Ad‐MSCs, and sampling time points. *, safety visits, 1 week before the next Ad‐MSC infusion. Ad‐MSC, adipose tissue allogeneic adult mesenchymal stromal cells

Ad‐MSCs were administered through a peripheral venous catheter over 1 to 2 hours using an infusion pump (infusion rate 2 mL/min) at a dose of 1 × 10^6^ Ad‐MSCs per kilogram of body weight at baseline and at weeks 4, 8, and 20 (Figure 1). Before administration, the cell suspension was tempered and stirred manually or using an electric stirrer to dissolve any cell aggregates that could have occurred during transport, and subjects received premedication with methyl prednisolone (0.5 mg/kg i.v.), dexchlorpheniramine (5 mg, i.v.), and oral acetaminophen.

### 2.5 Study subjects

The subjects were selected among the HIV‐infected adults followed at the Virgen del Rocío University Hospital. An INR was defined as an HIV‐infected subject with a basal CD4^+^ T‐cell count ≤350 cells per microliter whose CD4^+^ T‐cell count increased <75 or <150 cells per microliter after 1 or 2 years with undetectable viral load, respectively, and/or a CD4^+^ T‐cell count increase <350 cells per microliter after 3 years on treatment. Exclusion criteria included opportunistic infections in the previous 12 months, active coinfections with hepatitis B virus or hepatitis C virus, Child‐Pugh class C liver cirrhosis, portal hypertension or hypersplenism, malignant tumors, or treatment in the previous 12 months with immunomodulators, interferon, chemotherapy, or any other drug that might alter CD4^+^ T‐cell count. Pregnant or breastfeeding women and subjects refusing to use accepted contraceptive methods throughout follow‐up were also excluded from participation in the trial. All subjects agreeing to participate in the clinical trial provided written informed consent before undergoing any study‐related procedure.

### 2.6 Endpoints, follow‐up, and assessments

The main aims of the study were to assess the safety and efficacy of four infusions of Ad‐MSCs. In accordance with GCP, all adverse events (AEs) observed by the investigator or reported by the subjects, whether or not attributed to the investigational medicinal product (IMP), were carefully monitored and recorded. AEs were summarized and classified on the basis of MedDRA terminology and their relationship to Ad‐MSC administration and categorized via the standardized toxicity‐grade scale used by the AIDS Clinical Trials Group.^17^ The causality of AEs with the IMP was assessed by the principal investigator and reevaluated by a qualified person responsible for pharmacovigilance appointed by the trial's sponsor.

Efficacy was measured as the changes in CD4^+^ T‐cell counts, percentage of CD4^+^ T cells, and CD4^+^/CD8^+^ ratios after infusions and throughout 96 weeks after the first Ad‐MSC dose. The subjects had a total of 12 visits: at baseline and on weeks 3, 4, 7, 8, 12, 19, 20, 24, 36, 48, and 96, when adverse events, adherence to ART (subjects' self‐report and pharmacy records), hematology and chemistry tests, CD4^+^ and CD8^+^ T‐cell counts, and plasma HIV‐RNA levels were assessed. The CD4^+^ and CD8^+^ T‐cell counts were determined in fresh blood with an FC 500 flow cytometer (Beckman Coulter, Brea, CA). Plasma HIV‐1 RNA levels were measured by quantitative polymerase chain reaction (Cobas AmpliPrep‐Cobas TaqMan HIV‐1 test, version 2.0; Roche Diagnostics, Basel, Switzerland) with a lower detection limit of 20 copies per milliliter, according to the manufacturer's instructions.

### 2.7 Laboratory measurements

#### 2.7.1 Cell surface staining for immune profile

Peripheral blood mononuclear cells (PBMCs) were isolated using a gradient technique with Ficoll and stored with fetal bovine serum and 10% dimethyl sulfoxide in liquid nitrogen until assay time. On the day of the assay, frozen PBMCs were thawed at 37°C and washed with phosphate‐buffered saline. Afterward, cells were stained with different fluorochrome‐conjugated antibodies to assess the expression of surface markers, including CD3‐BV786, CD4‐APC‐Cy7, CD8‐BV510, CD45RA‐Pe‐Cy7, CD27‐BV421, HLA‐DR‐PE‐CF594, Lineage Cocktail 1‐FITC, CD56‐PE‐CF594, CD16‐AF700, CD25‐BV421, Foxp3‐PE, PD1‐BV605, Ki67‐FITC, CD38‐BV605, HLA‐DR‐FITC, CD57‐APC, CD28‐PE, Annexin V‐APC, and CD31‐PE (all from BD Biosciences, San Jose, CA) and CD11c‐PE and CD123‐APC (BioLegend, San Diego, CA). Viable cells were identified using 7AAD‐PerCP‐Cy5.5 (BD Biosciences) or LIVE/DEAD fixable Yellow Dead Cell Stain (Invitrogen, Carlsbad, CA) for intracellular staining. The cellular markers were analyzed by multicolor flow cytometry on total CD4^+^ (CD3^+^ CD4^+^) and CD8^+^ (CD3^+^ CD8^+^) T cells, and the different subsets were defined as follows: naïve (T_N_) CD45RA^+^ CD27^+^, CD4^+^ recent thymic emigrants (RTEs) CD45RA^+^ CD27^+^ CD31^+^, central memory (T_CM_) CD45RA^**−**^ CD27^+^, effector memory (T_EM_) CD45RA^**−**^ CD27^**−**^, and terminally differentiated (T_EMRA_) CD45RA^+^ CD27^**−**^. Other cellular subsets were defined as follows: myeloid dendritic cells (mDCs; Lin‐1^−^ HLA‐DR^+^ CD11c^+^ CD123^**−**^), plasmacytoid dendritic cells (pDCs; Lin‐1^−^ HLA‐DR^+^ CD11c^−^ CD123^+^), NK cells (CD3^−^ CD56^+^ CD16^+^), and T_reg_ cells (CD3^+^ CD4^+^ CD25^++^ Foxp3^+^). Moreover, the frequency of T cells expressing markers of cellular proliferation (Ki67), apoptosis (Annexin V), exhaustion (PD‐1), activation (HLA‐DR/CD38), and replicative senescence (CD28^−^ CD57^+^) were also quantified in both total CD4^+^ and CD8^+^ T cells. Samples were acquired on a Fortessa LSR II instrument (BD Biosciences, Madrid, Spain), discarding dead cells, and analyzed using FlowJo 9.3.2 software.

#### 2.7.2 Cellular associated HIV‐DNA

Total cellular associated HIV‐DNA (integrated and unintegrated viral DNA) extracted from PBMCs using Blood DNA Mini Kit (Omega Bio‐Tek, Norcross, GA) was assayed by real‐time polymerase chain reaction using specific primers (forward: 5′‐TAGCGGAGGCTAGAAGGAGA‐3′; reverse: 5′‐CCTGGCCTTAACCGAATT‐3′) and a probe within the selected gag long terminal repeat region (5′‐TACCGACGCTCTCGCACCCA‐3′) labeled with FAM.

#### 2.7.3 Enzyme‐linked immunosorbent assays

Plasma samples were aliquoted and stored at −20°C until subsequent analysis of the following biomarkers: high‐sensitivity C‐reactive protein (hsCRP), interleukin (IL)‐6, TNF‐α, and soluble CD14 (sCD14). The levels of hsCRP were determined with an immunoturbidimetric serum assay using Cobas 701 (Roche Diagnostics, Mannheim, Germany). Commercially available enzyme‐linked immunosorbent assays were used for the assay of IL‐6 (Quantikine HS Human IL‐6 immunoassay kit; R&D Systems, Minneapolis, MN), TNF‐α (using Quantikine HS Human TNF‐α immunoassay kit; R&D Systems), and sCD14 (Human CD14 ELISA Kit; Thermo Fisher Scientific, Waltham, MA) following the manufacturers' instructions.

### 2.8 Statistical analysis

Results were expressed as median values with interquartile ranges (IQRs) for continuous variables and as numbers and percentages of cases for categorical variables. The Wilcoxon signed rank test was performed to compare changes in continuous variables over time. Differences were considered statistically significant if the *P* value was <.05. The statistical analyses were performed using IBM software (SPSS, version 23.0; SPSS Inc., Chicago, IL).

## 3 RESULTS

### 3.1 Characteristics of the participants

Five White INRs were included in the initial phase of the study. After evaluating the results at week 48, an IDMC recommended the suspension of the clinical trial. Therefore, here we report the results of these five subjects. All of them were men and completed the study with an ART adherence of 100%. Overall, the median (IQR) basal age was 53 (45‐58) years, CD4^+^ T‐cell nadir 16 (2‐108) cells per microliter, CD4^+^ T‐cell count 253 (211‐340) cells per microliter, CD4^+^/CD8^+^ T‐cell ratio 0.42 (0.19‐0.48), percentage of CD4^+^ T cells 24.1% (11.4‐z25.5), months on ART 109 (73‐210), and 69 (53‐84) months of continuous undetectable plasma HIV‐RNA levels before their inclusion in the study (Table 1).

**TABLE 1:** Baseline characteristic of the subjects

Patient number	Age (years)	BMI (kg/m^2^)	HIV transmission route	Smoker	CDC stage	Months on treatment	HIV‐RNA <50 copies/mL (months)	Nadir CD4^+^ count/μL	CD4^+^ count/μL	% CD4^+^	CD4^+^/CD8^+^
1	47	31.5	IVDU	Yes	A3	109	52	3	269	24.10	0.46
2	56	33.7	HTS	Yes	C3	184	69	16	251	11.44	0.20
3	53	18.0	IVDU	Yes	C3	237	77	2	171	11.49	0.19
4	61	27.4	HTS	No	C3	93	91	37	412	24.30	0.42
5	43	30.8	HTS	Yes	A3	54	53	180	253	26.70	0.50

Abbreviations: BMI, body mass index; CDC, Centers for Disease Control and Prevention; HTS, heterosexual contact; IVDU, previous intravenous drug use.

### 3.2 Safety and tolerability of Ad‐MSC infusions in HIV‐infected subjects

In total, 10 venous thrombosis events, 24 to 48 hours after Ad‐MSC infusion, were observed in three out of the five subjects in the arms where Ad‐MSCs were infused, which required low molecular weight heparin treatment. A decrease in the emergence of thrombotic events was observed when low molecular weight heparin (80 mg of enoxaparin per day) was given 1 day before, on the day of the infusion, and during the following 2 days, together with a retraining course on the cell infusion procedure, as it was detected that the infusion rate was significantly higher than that established by protocol in one case. All other adverse events (n = 5) were considered unrelated to the Ad‐MSCs (Table 2). Furthermore, no subjects presented alterations in the biochemical and hematological parameters or increases in HIV viral load (data not shown).

**TABLE 2:** MedDRA coded (version 22.1) adverse events occurred within 96 weeks of follow‐up

MedDRA: System organ class/preferred term	Number of episodes	Number of cases	Relation with IMP
Administration site reactions	10	3	Y
Infusion site thrombosis	10		
Infections and infestations	6	3	N
Nasopharyngitis	1		
Conjunctivitis	1		
Herpes ophthalmic	1		
Pneumonia	1		
Diarrhea	1		
Tonsillitis	1		
General disorders and administration site conditions	2	2	N
Pyrexia	2		
Injury, poisoning and procedural complications	2	2	N
Trauma	1		
Accident	1		
Psychiatric disorders	1	1	N
Anxiety	1		
Gastrointestinal disorders	1	1	N
Hiatal hernia	1		
Respiratory, thoracic, and mediastinal disorders	1	1	N
Dyspnea	1		
Skin and subcutaneous tissue disorders	1	1	N
Pruritus	1		
Nervous system disorders	1	1	N
Syncope	1		
Renal and urinary disorders	1	1	N
Microalbuminuria	1		

Abbreviations: IMP, investigational medicinal product; N, no; Y, yes.

### 3.3 Efficacy of Ad‐MSC infusions in HIV‐infected subjects

**FIGURE 2:**
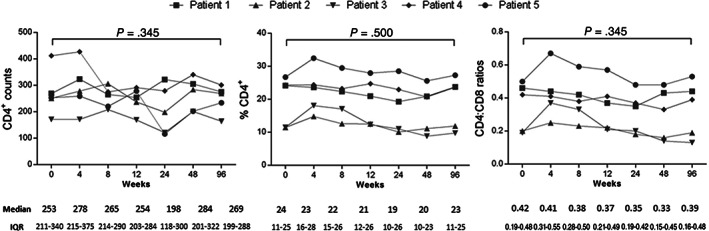
Evolution of the CD4^+^ T‐cell counts, percentages, and CD4^+^/CD8^+^ ratios after 96 weeks of follow‐up. IQR, interquartile range

Overall, no significant changes were observed in the CD4^+^ T‐cell counts, percentage of CD4^+^, or CD4^+^/CD8^+^ ratios after infusions and throughout follow‐up (Figure 2). Likewise, there were no significant changes in the different subsets of CD4^+^ and CD8^+^ T lymphocytes (T_N_, RTE, T_CM_, T_EM_, and T_EMRA_) except for an increase in the T_EM_ CD8^+^ T subset (Figures S3 and S4); nor were there changes in the percentage of NK cells, T_reg_ cells, mDCs, and pDCs (Figure S5). Furthermore, we did not observe changes in the activation, proliferation, senescence and apoptosis, or exhaustion markers of CD4^+^ or CD8^+^ T cells. We observed a decrease in the percentage of PD1^+^ CD4^+^ T cells (5.2 vs 1.6; *P* = .043) and a trend in PD1^+^ CD8^+^ T cells (0.9 vs 0.4; *P* = .080) at week 96 (Figures S6 and S7). On the other hand, the sCD14 plasma levels, measured with monocyte activation markers and the different proinflammatory proteins (IL‐6, TNF‐α, hsCRP) showed no significant changes (Figure S8). Likewise, the viral reservoir, measured as total cell‐associated HIV‐DNA, remained stable throughout the follow‐up (Figure S9).

## 4 DISCUSSION

Among the different properties of MSCs, their immunoregulatory potential is noteworthy because they can interact with cells of both the innate and adaptive immune systems, leading to the modulation of several effector functions.^18^, ^19^

MSCs are able to suppress T lymphocyte activation and proliferation by decreasing the production of TNF‐α and IFN‐γ while inducing IL‐10 and IL‐4 expression by CD4^+^ T cells. In addition, they inhibit the dendritic cell maturation and natural killer cell activation, induce T_reg_ cell differentiation, and promote a shift of macrophage toward anti‐inflammatory phenotype, as well as secrete anti‐inflammatory cytokines such as IL‐1Ra, IL‐10, TGF‐β, and hepatocyte growth factor, among others.^14^, ^18^, ^20^

Moreover, given their low immunogenicity, both autologous and allogeneic cells can safely be administered.^21^, ^22^, ^23^, ^24^

Zhang *et al*.^16^ have reported the only study carried out with MSCs in HIV‐infected subjects in which they transfused umbilical cord MSCs at a dose of 0.5 × 10^6^ cells per kilogram body weight on day 0, month 1, and month 2 to seven INRs. They observed neither short‐term clinical adverse effects nor HIV‐1 load rebound, and six of the seven subjects displayed a sharp increase in CD4^+^ T‐cell counts of more than 50% compared with baseline values. In this study the umbilical cord MSCs preferentially expanded naïve and central memory CD4^+^ T‐cell counts, whereas the effector memory and the terminally differentiated subsets were gradually decreased. Moreover, Zheng *et al*. observed a significant decrease in the percentages of CD38^+^ and CD38^+^ HLA‐DR^+^ CD8 T cells, decreased PD‐1 expression on total CD4^+^ and CD8^+^ T cells, and a significant reduction in plasma levels of several proinflammatory cytokines and chemokines.

In our study, the subjects received four doses (1 × 10^6^/kg) of Ad‐MSCs, but no changes were observed in the CD4^+^ T‐cell counts, percentages, or CD4^+^/CD8^+^ ratios, and no consistent changes were observed in the different subsets and phenotypes of the CD4^+^ and CD8^+^ T cells or percentage of mDCs, pDCs, NK cells, or T_reg_ cells. Likewise, the infusions of Ad‐MSCs had no effects on sCD14, IL‐6, TNF‐α, and hsCRP plasma levels. Overall, we did not find a decrease in activation, exhaustion, apoptosis, and senescence at week 96. Only a significant decrease in the PD1^+^ CD4^+^ T cells was found, but its meaning is uncertain, and this change did not influence CD4^+^ T‐cell recovery.

The principal differences with our study are the origin of the MSCs and the doses administered. Whereas Zhang *et al*.^16^ obtained MSCs from umbilical cord, specifically Wharton's jelly, we used adipose tissue, which is a more accessible source for obtaining MSCs. Although MSC immunoregulatory properties are unrelated to their origin (bone marrow, adipose tissue, or umbilical cord),^25^, ^26^ we are not certain if the different cellular origin could be responsible for the discrepant results. Adult MSCs offer numerous advantages, but it has been reported that the yield, proliferative potential, and plasticity of MSCs decreases progressively with the advancing age of the donor compared with embryonic stem cells.^27^ Also, Donders *et al*. showed that genes associated with cell adhesion, proliferation, and modulation of the immune system are enriched in Wharton's jelly‐derived MSCs.^28^

Likewise, recently, it has been proposed that other factors, such as tobacco, diabetes, or morbid obesity, can influence not only cell performance during manufacturing but also their pharmacological action.^29^, ^30^ All this could have influenced the lack of efficacy of MSCs in our study.

The high number of venous thrombosis events (VTEs) observed could have a complex multifactorial causality. On one side, the study subjects may be at increased risk of VTEs because of the patients' history of parenteral drug use (2/5), higher risk of recurrence in cases of previous thrombotic episodes,^31^, ^32^ or a high infusion rate (>3 mL/min) recorded in some infusions. In fact, the total of 10 events occurred in only three subjects, all having received four doses of the Ad‐MSC suspension. Furthermore, in one patient, the VTEs could have been caused by the use of small‐caliber veins on the back of the hands for the infusions, as this patient could not be infused in the arm. In addition, it has been reported that MSCs express tissue factor and have procoagulant activity, being observed more frequently in Ad‐MSCs than in those of other sources. Furthermore, cell dose, handling conditions, growth media, and donor‐associated factors might also influence procoagulant activity.^33^, ^34^, ^35^, ^36^

Our study has several limitations. Regarding the safety of Ad‐MSCs, it would have been desirable to determine their tissue factor expression before their administration, although in more than 150 patients treated with this same product in different clinical trials sponsored by the Andalusian Network for the Design and Translation of Advanced Therapies, thrombotic events occurred exceptionally. Furthermore, a full characterization of the infused MSCs was carried out, but neither in vitro functional assay to determine their immunomodulatory potency nor biodistribution analysis was performed. However, previous studies have shown that after intravenous infusion, cells were accumulated in lung, spleen, liver, and bone marrow.^37^

On the other hand, a higher number of patients and a control group, planned in the second phase of the trial, would have allowed us to better assess the fluctuations that some patients presented in several immunological parameters. Furthermore, the analyses are based on PBMC samples, which may not always reliably reflect tissue‐related processes during HIV infection. The use of lymph node samples instead of PBMCs would have needed periodical biopsies, lowering the feasibility of the study.

## 5 CONCLUSION

The Ad‐MSC infusions have not proven to be effective to improve the immune recovery, nor have they succeeded in reducing immune activation or levels of inflammatory markers in INRs, at least with the dosage schedule selected.

## CONFLICT OF INTEREST

The authors declared no potential conflicts of interest.

## AUTHOR CONTRIBUTIONS

M.T.‐R.: collection and/or assembly of data, data analysis and interpretation, manuscript writing, final approval of manuscript; P.V., A.R.‐G., O.E.‐I., S.A.‐S.: provision of study material or patients, final approval of manuscript; I.R.‐J.: administrative support, collection and/or assembly of data, final approval of manuscript; A.I.Á.‐R.: data analysis and interpretation, final approval of manuscript; J.M.‐A., R.M., O.F.‐L.: financial support, administrative support, final approval of manuscript; E.R.‐M.: data analysis and interpretation, final approval of manuscript; A.G.‐V.: conception/design, data analysis and interpretation, manuscript writing, final approval of manuscript; L.F.L.‐C.: conception/design, provision of study material or patients, final approval of manuscript.

## Supporting information


**Data S1.** Supporting Information.

## Data Availability

The data that support the findings of this study are available on request from the corresponding author.
