# Supplementary material for: Mesenchymal stromal cells in human immunodeficiency virus‐infected patients with discordant immune response: Early results of a phase I/II clinical trial
Source: Stem Cells Transl Med. 2020 Dec 2;10(4):534–41. doi: 10.1002/sctm.20-0213 (PMC7980217; doi:10.1002/sctm.20-0213)
Supplement: Supplementary file 1 — Data S1. Supporting Information. [file SCT3-10-534-s001.pdf]

Supplementary Table 1. Clinical information about donors

PATIENT	DOSE	BATCH	DONOR	DIABETES	HYPERTENSION	SMOKER	IMC
EC19/D/01	1	1605D05	1605	YES	NO	YES	37,5
	2	1605D06					
	3	1605D09					
	4	1701D10	1701	NO	NO	NO	45,88
EC19/D/02	1	1605D07	1605	YES	NO	YES	37,5
	2	1607D04	1607	NO	NO	NO	32,71
	3	1607D08					
	4	1701D13	1701	NO	NO	NO	
EC19/D/03	1	1607D03	1607	NO	NO	NO	32,71
	2	1701D03	1701	NO	NO	NO	45,88
	3	1701D06					
	4	1701D16					
EC19/D/04	1	1701D08	1701	NO	NO	NO	45,88
	2	1701D11					
	3	1701D12					
	4	1701D18					
EC19/D/05	1	1701D17	1701	NO	NO	NO	45,88
	2	1702 D03	1702	NO	NO	NO	Not available
	3	1702 D05					
	4	1702 D06					

Supplementary Table 2. Quality controls after the expansion Ad-MSC.

		BACTH			
		**1605**	**1607**	**1701**	**1702**
**Viability (%)**		97,0	92,4	97,7	95,2
**Sterility test**		Absence of microorganisms	Absence of microorganisms	Absence of microorganisms	Absence of microorganisms
**Gram stain**		Absence of microorganisms	Absence of microorganisms	Absence of microorganisms	Absence of microorganisms
**Calcofluor stain**		Absence of fungal structures	Absence of fungal structures	Absence of fungal structures	Absence of fungal structures
**Mycoplasma**		Absence of mycoplasma	Absence of mycoplasma	Absence of mycoplasma	Absence of mycoplasma
**Cariotype**		Absence of chromosomal abnormalities	Absence of chromosomal abnormalities	Absence of chromosomal abnormalities	Absence of chromosomal abnormalities
**Differentiation**	**Adipocytes**	Positive differentiation	Positive differentiation	Positive differentiation	Positive differentiation
	**Osteocytes**	Positive differentiation	Positive differentiation	Positive differentiation	Positive differentiation
**Phenotype**	**CD90**	99,50	98,98	100,00	98,60
	**CD73**	99,60	96,40	100,00	99,50
	**CD105**	100,00	98,98	100,00	98,60
	**CD 44**	100,00	98,05	97,00	97,55
	**CD45**	0,00	0,00	0,00	0,00
	**CD19**	0,00	0,00	0,00	0,00
	**CD11b**	0,00	0,00	0,00	0,00
	**HLA DR**	0,00	0,00	0,00	0,00
**DNA Fingerprint**		Matching donor	Matching donor	Matching donor	Matching donor
**Adventitious virus**		Absence of virus	Absence of virus	Absence of virus	Absence of virus

Supplementary Table 3. Quality control before delivery

PATIENT	DOSE	BATCH	VIABILITY	STERILITY	ENDOTOXINS	MYCOPLASMA
EC19/D/01	1	1605D05	96,50	Absence of microorganisms	<1,00	Absence of mycoplasma
	2	1605D06	95,20	Absence of microorganisms	<1,00	Absence of mycoplasma
	3	1605D09	98,00	Absence of microorganisms	<1,00	Absence of mycoplasma
	4	1701D10	95,91	Absence of microorganisms	<1,00	Absence of mycoplasma
EC19/D/02	1	1605D07	97,20	Absence of microorganisms	<1,00	Absence of mycoplasma
	2	1607D04-D05	96,20	Absence of microorganisms	<1,00	Absence of mycoplasma
	3	1607D08	97,80	Absence of microorganisms	<1,00	Absence of mycoplasma
	4	1701D13	96,10	Absence of microorganisms	<1,00	Absence of mycoplasma
EC19/D/03	1	1607D03	98,00	Absence of microorganisms	<1,00	Absence of mycoplasma
	2	1701D03	97,80	Absence of microorganisms	<1,00	Absence of mycoplasma
	3	1701D06	95,48	Absence of microorganisms	<1,00	Absence of mycoplasma
	4	1701D16	99,10	Absence of microorganisms	<1,00	Absence of mycoplasma
EC19/D/04	1	1701D08	98,70	Absence of microorganisms	<1,00	Absence of mycoplasma
	2	1701D11	98,75	Absence of microorganisms	<1,00	Absence of mycoplasma
	3	1701D12	95,80	Absence of microorganisms	<1,00	Absence of mycoplasma
	4	1701D18	98,40	Absence of microorganisms	<1,00	Absence of mycoplasma
EC19/D/05	1	1701D17	97,50	Absence of microorganisms	<1,00	Absence of mycoplasma
	2	1702 D03	98,40	Absence of microorganisms	<1,00	Absence of mycoplasma
	3	1702 D05	97,60	Absence of microorganisms	<1,00	Absence of mycoplasma
	4	1702 D06	96,70	Absence of microorganisms	<1,00	Absence of mycoplasma

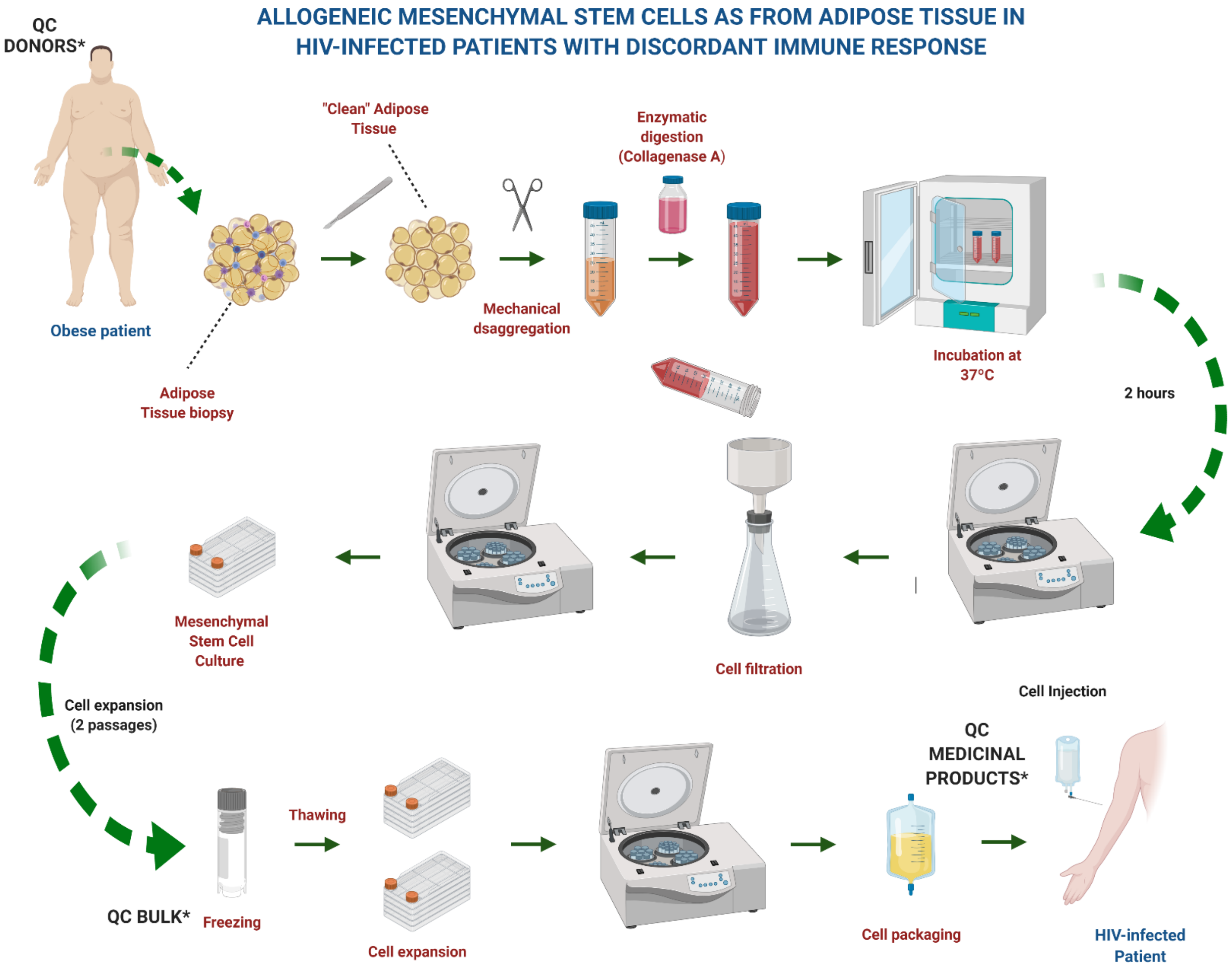

Supplementary Figure 1. An overview of the manufacturing process of allogeneic mesenchymal stem cells.

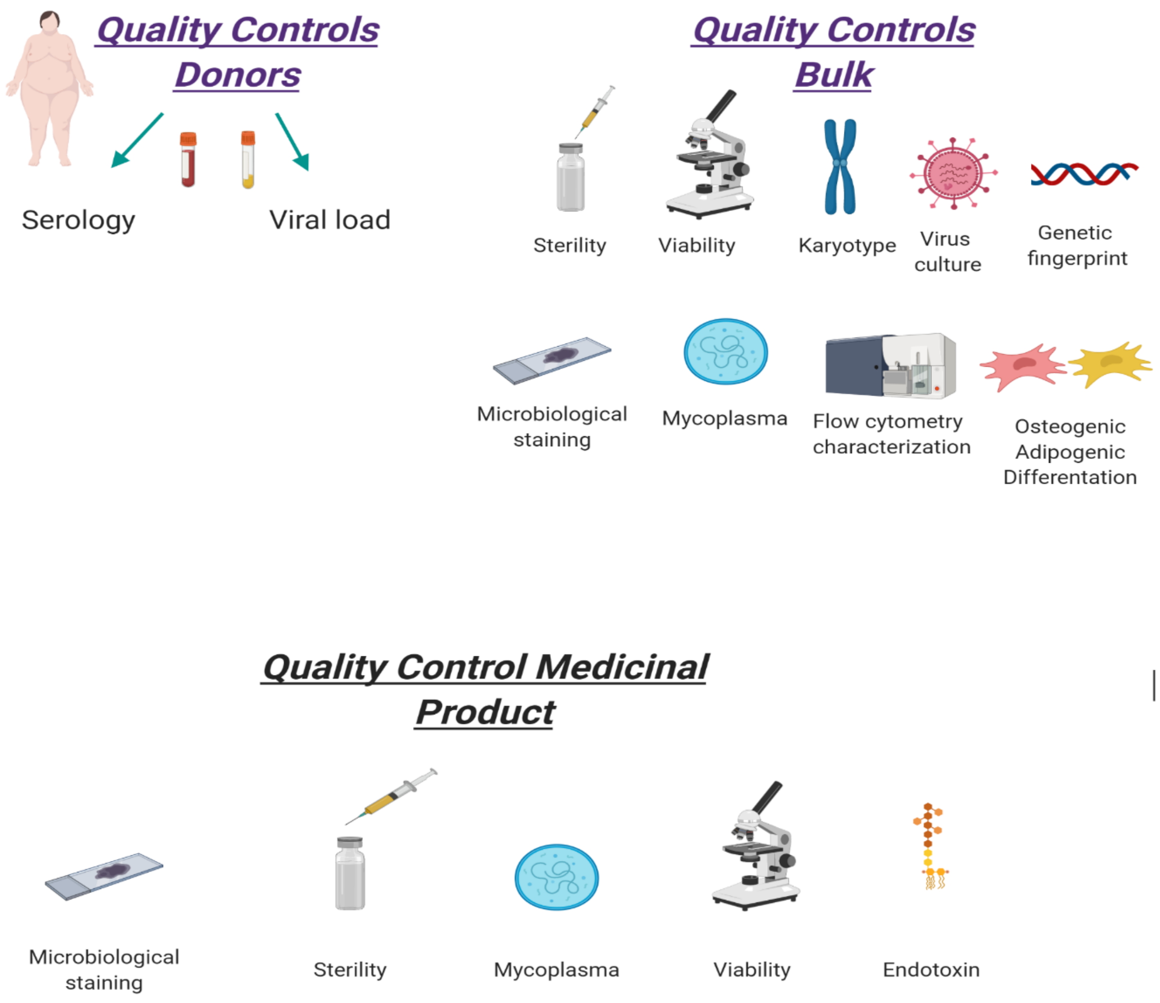

Supplementary Figure 2. Quality controls of mesenchymal stem cells.

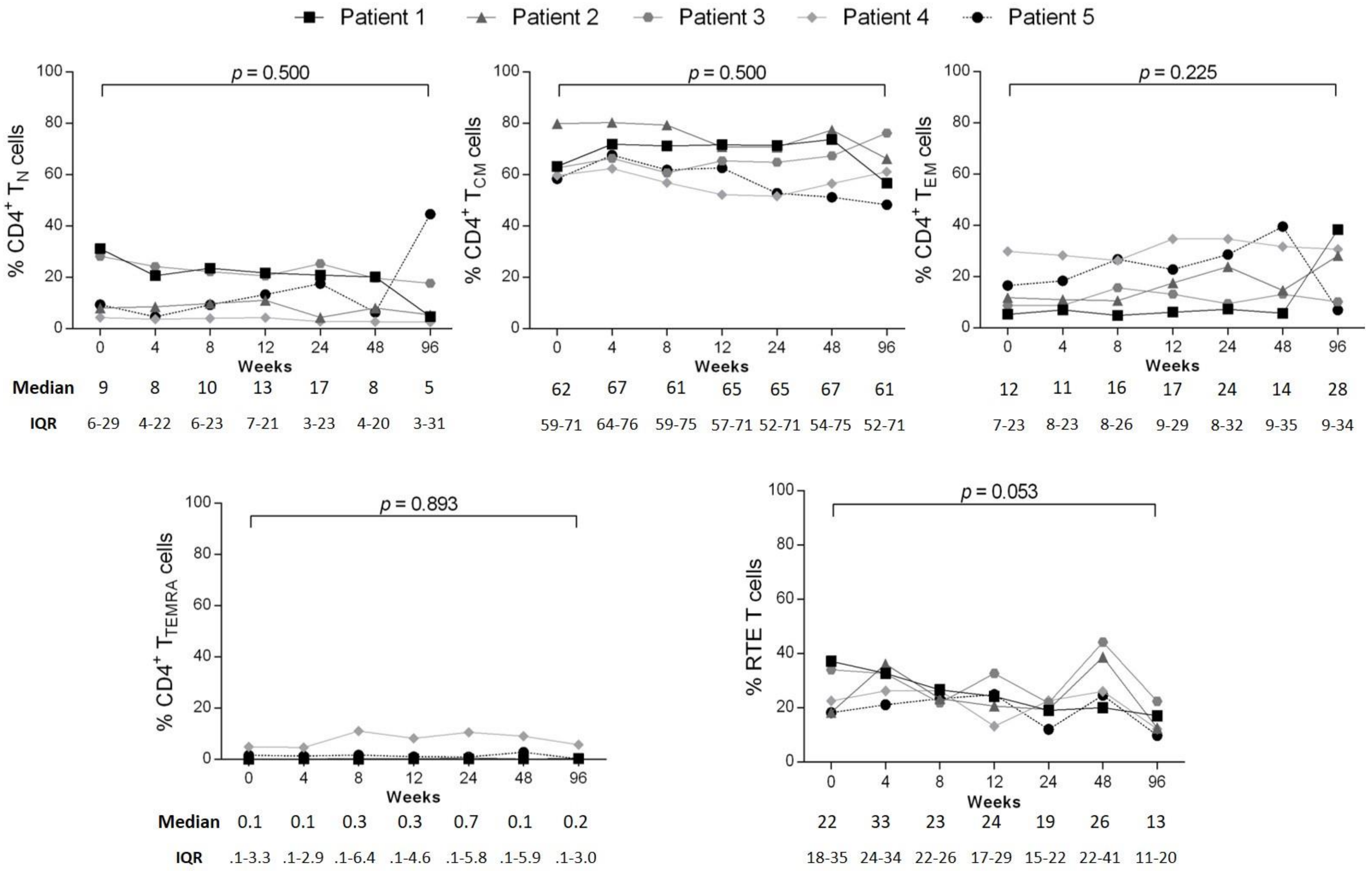

Supplementary Figure 3. Evolution of the percentage of $CD4^+$ T cell subsets through 96 weeks of follow-up. N, naïves. CM, central memory. EM, effector memory. TEMRA, terminally differentiated effector memory cells re-expressing CD45RA. RTE, recent thymic emigrants.

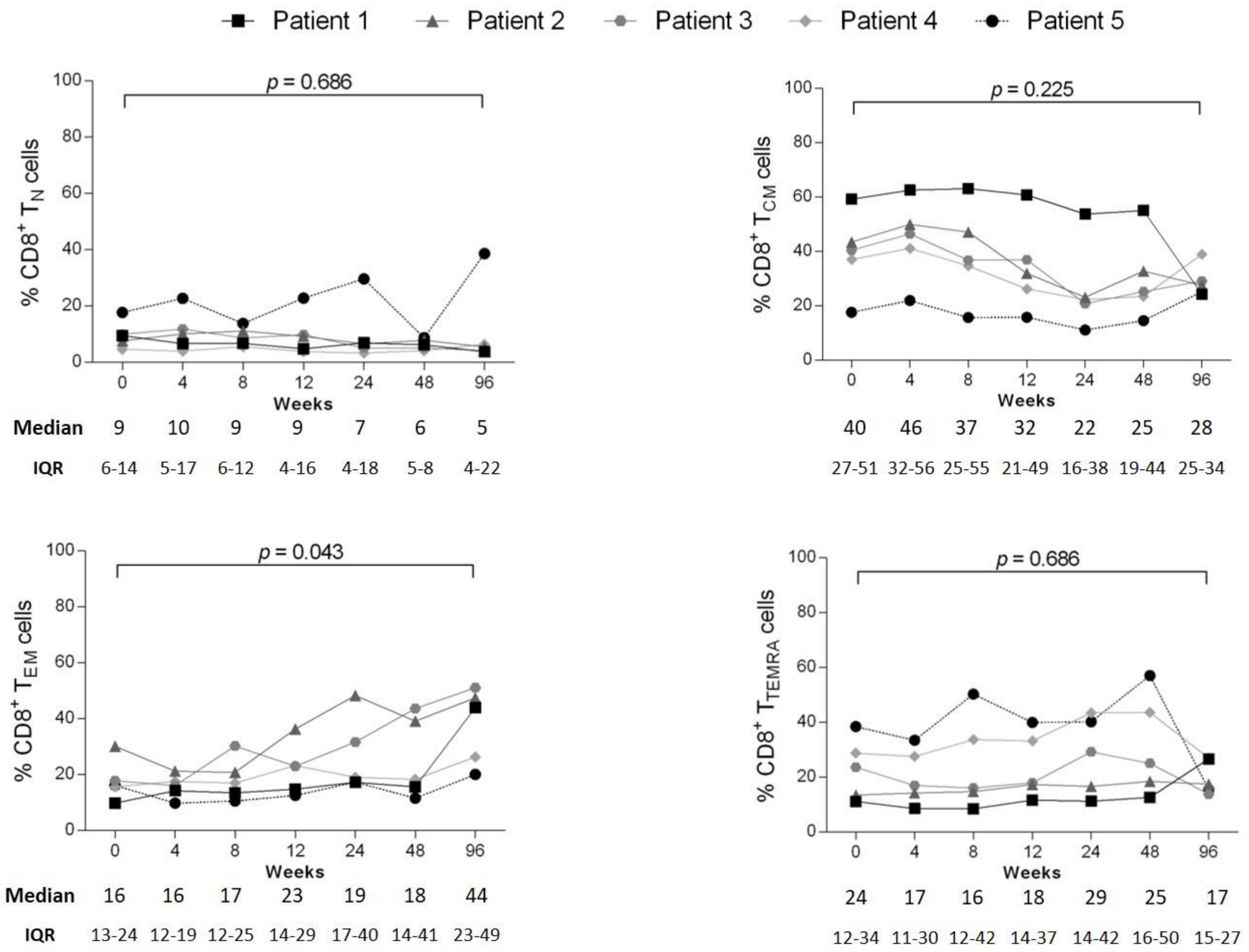

Supplementary Figure 4. Evolution of the percentage of CD8$^+$ T cell subsets through 96 weeks of follow-up. N, naïves. CM, central memory. EM, effector memory. TEMRA, terminally differentiated effector memory cells re-expressing CD45RA.

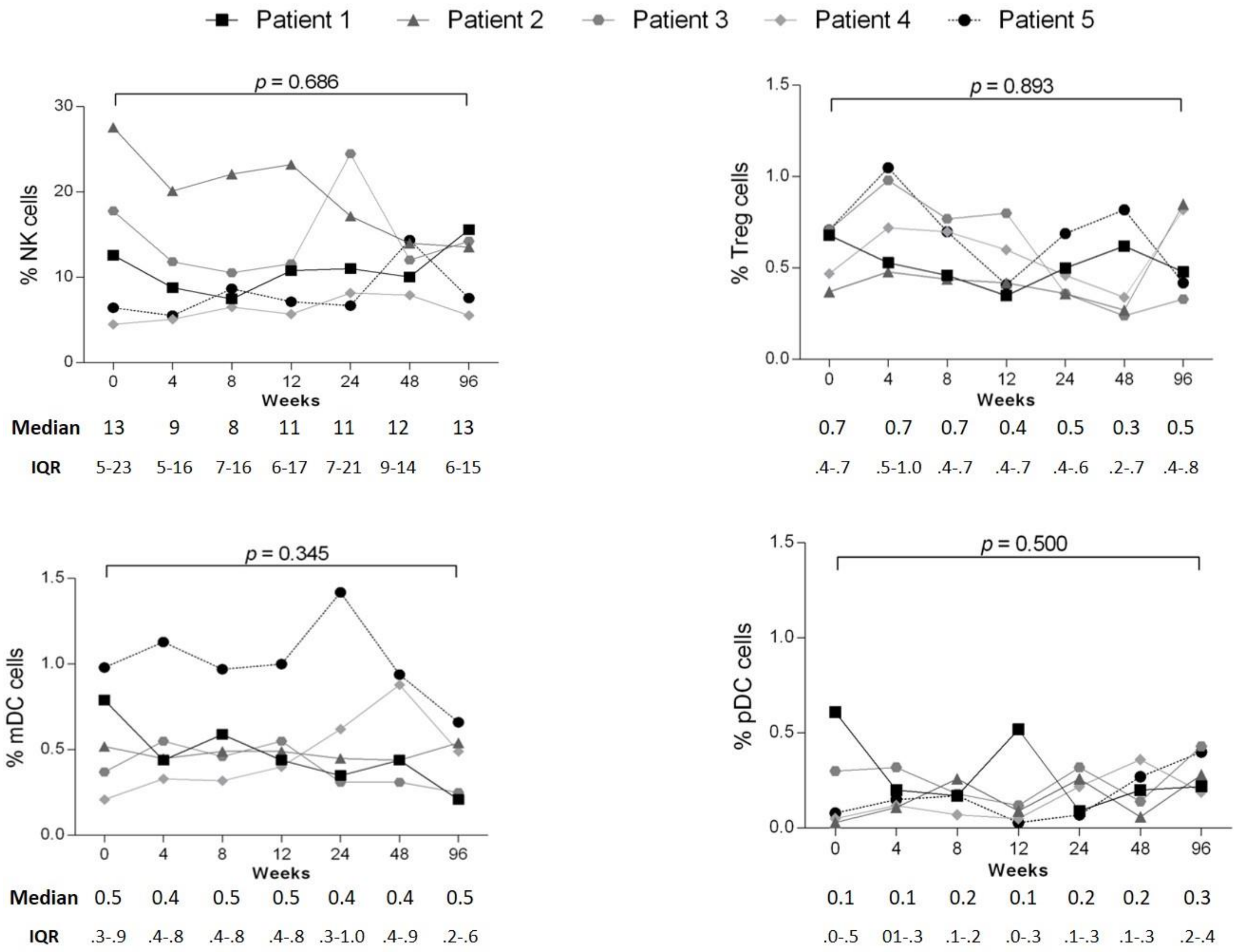

Supplementary Figure 5. Evolution of the percentage of Natural Killer Cells (NK), regulatory T-Cells (T_{regs}), myeloid (mDC) and plasmacytoid dendritic cells (pDC) through 96 weeks of follow-up.

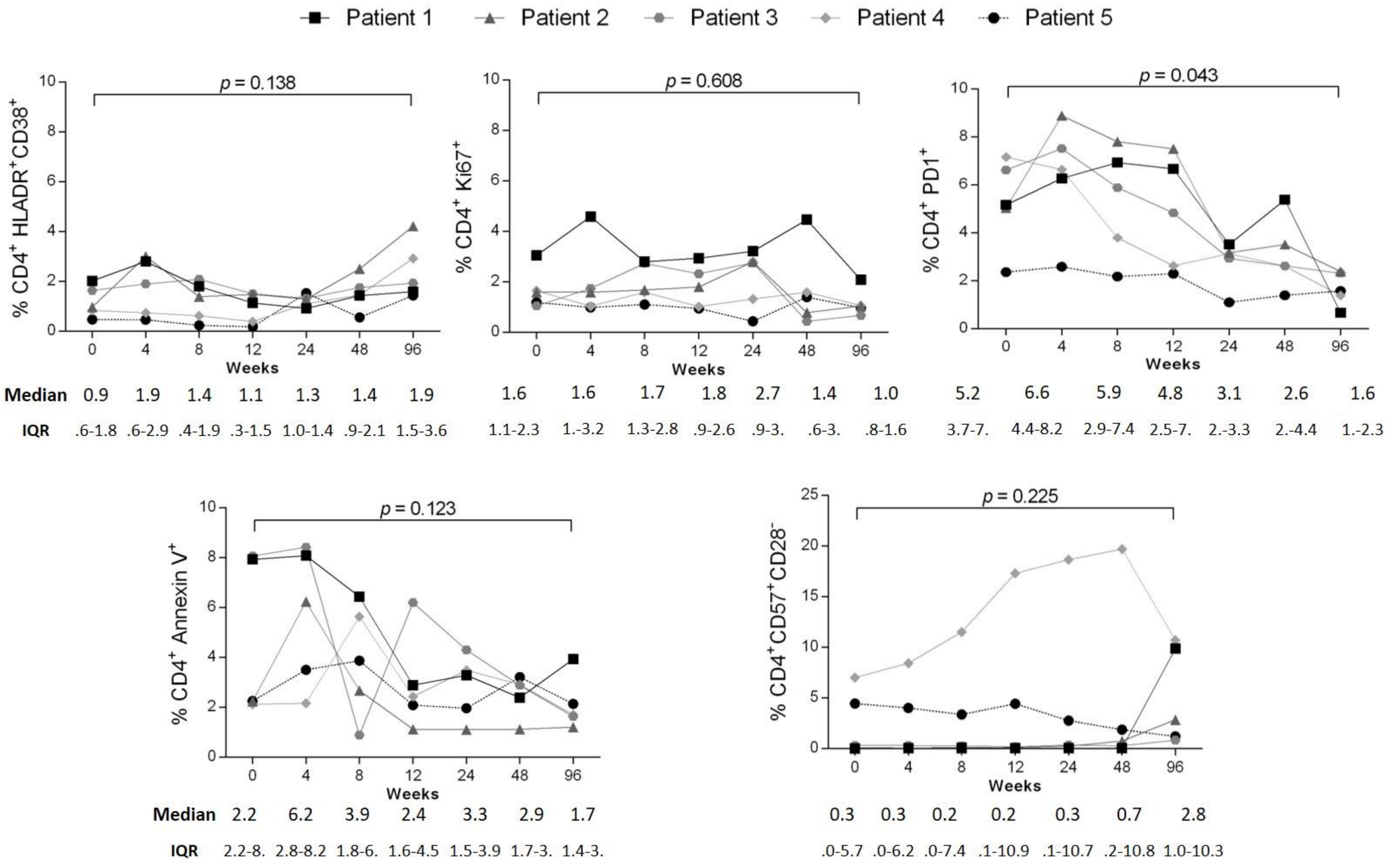

Supplementary Figure 6. Effect of mesenchymal stem cell transfusions in activation (HLA-DR$^+$CD38$^+$), proliferation (Ki67$^+$), exhaustion (PD1$^+$), apoptosis (annexin V$^+$) and senescence (CD57$^+$CD28$^-$) markers on CD4$^+$ T cells through 96 weeks of follow-up.

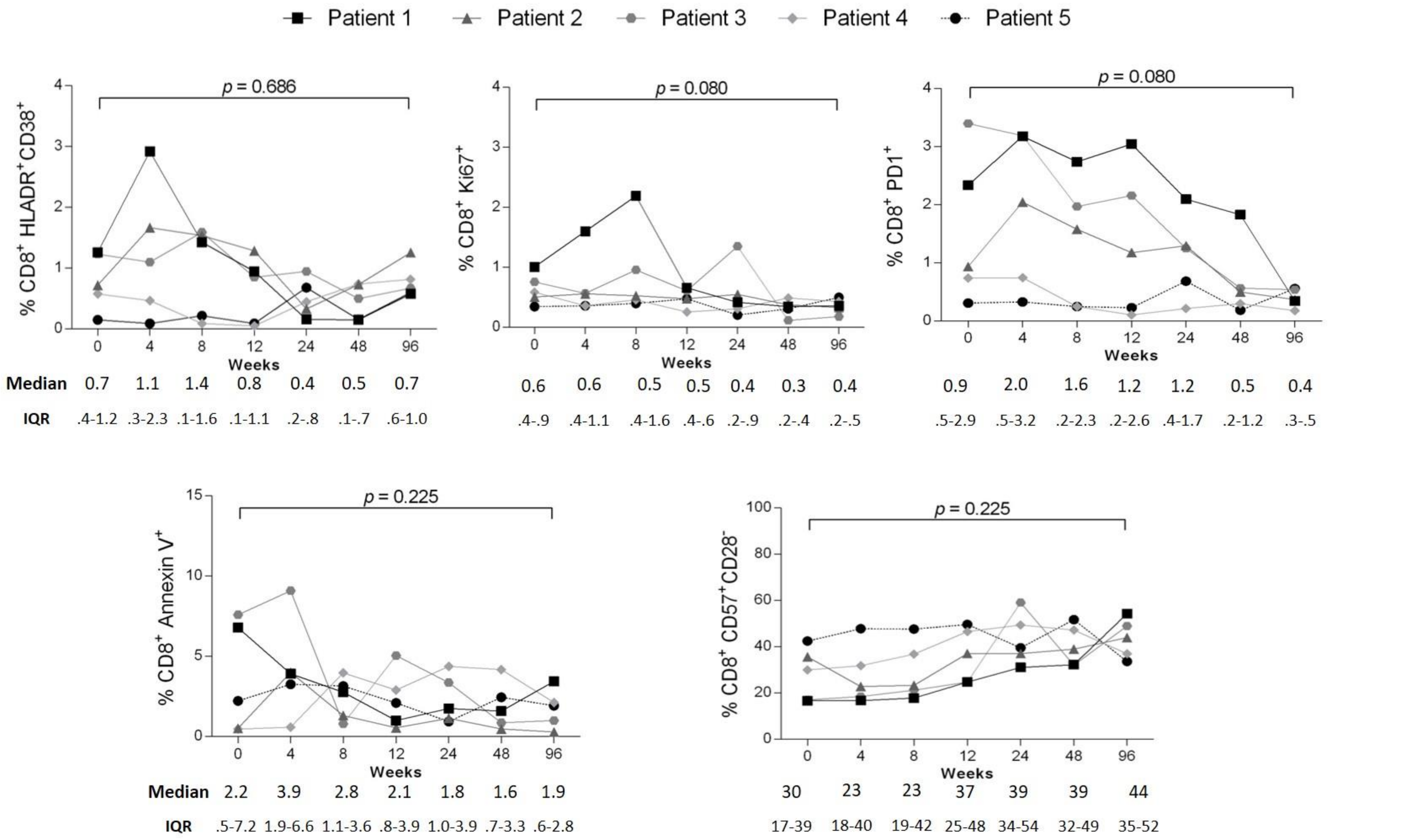

Supplementary Figure 7. Effect of mesenchymal stem cell transfusions in activation (HLA-DR^{+}CD38^{+}), proliferation (Ki67^{+}), exhaustion (PD1^{+}), apoptosis (annexin V^{+}) and senescence (CD57^{+}CD28^{-}) markers on CD8^{+} T cells through 96 weeks of follow-up.

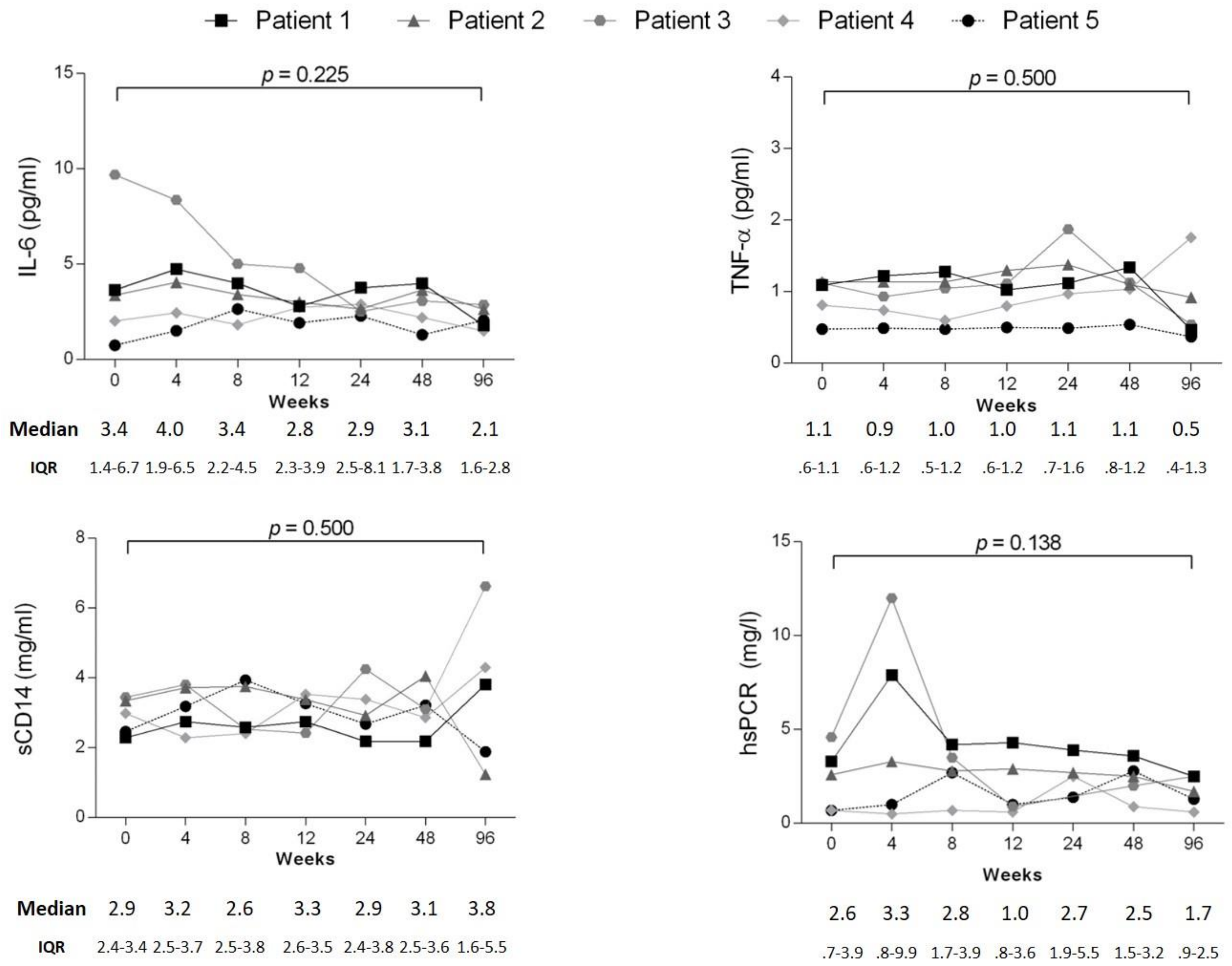

Supplementary Figure 8. Evolution of the plasma levels of interleukin 6 (IL-6), tumor necrosis factor-α (TNF-α), soluble CD14 (sCD14) and high sensitivity C-reactive protein (hsCRP) through 96 weeks of follow-up.

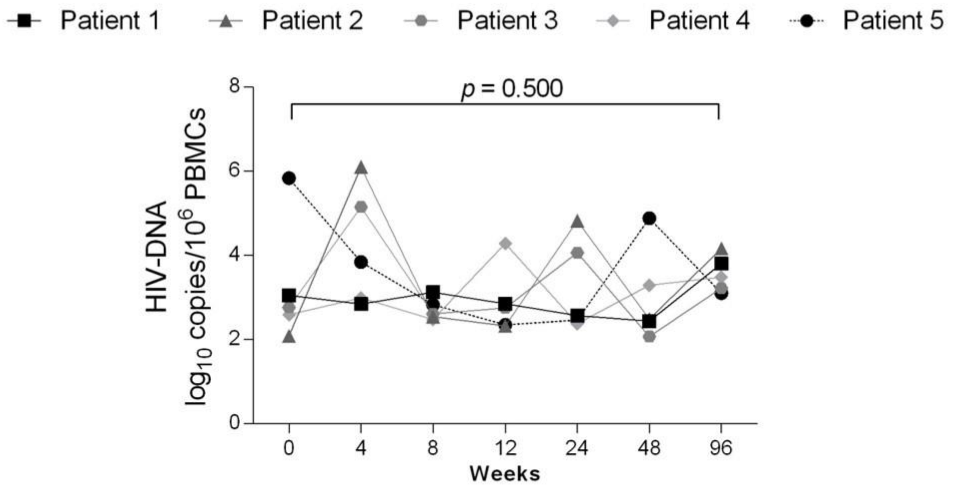

**Median**	2.8	3.0	2.6	2.8	2.6	2.5	3.5
**IQR**	2.3-4.4	2.7-4.5	2.5-2.9	2.3-3.6	2.4-4.4	2.3-4.1	3.2-3.9

Supplementary Figure 9. Evolution of cellular-associated HIV-DNA from PBMCs through 96 weeks of follow-up.
